# Experimental Myopia Results in Peripapillary Ganglion Cell and Astrocyte Reorganization with No Functional Implications During Early Development

**DOI:** 10.3390/ijms252413484

**Published:** 2024-12-16

**Authors:** Reynolds Kwame Ablordeppey, Carol Ren Lin, Miduturu Srinivas, Alexandra Benavente-Perez

**Affiliations:** Department of Biological Sciences, State University of New York College of Optometry, New York, NY 10036, USA; rablordeppey@sunyopt.edu (R.K.A.); clin@sunyopt.edu (C.R.L.); msrinivas@sunyopt.edu (M.S.)

**Keywords:** axial myopia, ganglion cell, astrocyte, retinal nerve fiber layer, marmoset, photopic negative response

## Abstract

Myopic eye growth induces mechanical stretch, which can lead to structural and functional retinal alterations. Here, we investigated the effect of lens-induced myopic growth on the distribution of retinal ganglion cells (RGCs), glial fibrillary acidic protein (GFAP) expression and intensity, and peripapillary retinal nerve fiber layer (ppRNFL) thickness in common marmosets (*Callithrix jacchus*) induced with myopia continuously for six months, using immunohistochemistry and spectral-domain optical coherence tomography. We also explored the relationship between cellular structural parameters and the photopic negative response (PhNR) using full-field electroretinography. Marmosets induced with myopia for six months developed axial myopia, had a thinner ppRNFL, reduced peripapillary ganglion cell (≈20%) and astrocyte density (≈42%), increased panretinal GFAP expression (≈42%) and nasal mid-periphery staining intensity (≈81%) compared to age-matched controls. Greater degrees of myopia and vitreous elongation were associated with reduced peripapillary RGCs and astrocyte density, and increased GFAP expression and intensity. These cellular structural changes did not show a significant relationship with the features of the PhNR, which remained unchanged. The outcomes of this study suggest that myopia induces a reorganization of the peripapillary inner retina at the cellular level that may not result in measurable functional repercussions at this stage of myopia development.

## 1. Introduction

Myopia (nearsightedness) represents a major public health challenge due to its increased risk of developing associated eye complications such as myopic maculopathy and glaucoma among others, which are related to the degree of myopia experienced [1]. Myopia has structural and functional consequences on the retina, possibly due to the sustained mechanical stress that myopic eye growth has on the retinal tissue [2]. However, the cellular mechanisms that make myopia a risk factor for blinding ocular conditions remain unknown. In addition, fully corrected myopic eyes can experience a reduction in visual performance compared to emmetropes, including lower resolution acuity [2,3] and contrast sensitivity [4]. Chui et al. [2] and Atchison et al. [3] proposed that this reduced visual performance may result from retinal stretching along with retinal ganglion cell (RGC) death or dysfunction. This hypothesis has not been investigated to date.

While RGCs may not be directly involved in the signaling process leading to myopia development [5], myopic growth does seem to have a significant impact on the structure and function of ganglion cells. Optical coherence tomography (OCT) reveals significant ganglion cell complex thinning in human [6,7] and animal models of myopia [8,9,10]. Chicks with induced myopia show a decline in the axonal diameter, degree of myelination and number of myelinated axons in the retinal nerve fiber layer (RNFL) [9], as well as a reduction in ganglion cell density, with a compensatory increase in ganglion cell dendritic arbors and branch lengths [11]. Most electroretinogram (ERG) recordings of inner retinal activity in humans show reduced amplitudes and delayed latencies of ERG components that are proportional to the degree of myopia and axial length, specifically full-field oscillatory potentials, N2 in multifocal, P50 and N95 in pattern, and the induced component of global-flash multi-focal ERGs [12,13,14,15,16].

Our lab has described relative inner retinal thinning, increased glial fibrillary acidic protein (GFAP) expression, reduced astrocyte density, reduced peripheral capillary branching and increased string vessel density in marmosets (*Callithrix jacchus*) induced with myopia compared to untreated controls [17,18]. The mild gliosis we have observed in myopic marmosets might precede or be secondary to ganglion cell alterations, and could be either beneficial or noxious for the viability and proper functioning of ganglion cells [19,20]. Furthermore, the reduced peripheral vascular density we identified may result in compromised RGC metabolic support, hypoxia, or oxidative stress in myopic eyes [21]. From the functional perspective, we have also identified an early reduction in the saturated amplitude of the photopic negative response (PhNR) in juvenile marmosets within two weeks of lens-induced myopia, before measurable eye growth changes occurred, which disappeared once marmosets developed axial myopia [22]. These point to underlying alterations in inner retinal anatomy or signal processing in myopia development and progression in marmosets.

While the effect of myopia on the gross anatomy and function of the retina has been studied in the past, whether potential cellular changes relate to functional alterations remains unknown. Among the few studies that have assessed retinal thickness and function in tandem, there is a lack of consensus regarding the nature of the relationship. There is evidence of retinal thinning along with a reduction in multi-focal ERG amplitudes and/or increased latency [23,24]. However, others report poor or no association between OCT structural measures and multi-focal ERGs [16,25]. In myopic chicks, an increase in near-ultraviolet reflectivity is associated with decreases in retinal nerve fiber and ganglion cell layer thickness, nerve fiber diameter and myelination [8,9]. Conversely, attenuated a-, b- and d-wave amplitudes from full-field ERGs have been reported to be independent of retinal structural changes in chicks with lens-induced myopia [26].

To date, how myopia affects ganglion cell density in non-human primate eyes with lens-induced myopia remains unknown. In this study, we provide a detailed characterization of the cellular changes observed in the ganglion cell complex (including RGC and astrocyte density, GFAP expression and intensity, and peripapillary RNFL thickness) in relation to myopic growth and inner retinal function using full-field PhNR in a non-human primate induced with myopia continuously for 6 months. Using a lens-induced myopia model, we aim to replicate the axial elongation process that underpins the development of natural myopia. The peripapillary RNFL was employed as a surrogate for panretinal RNFL since its measurement encompasses axons from all RGC bodies and contributes to the PhNR [27]. The findings from this study may help clarify the reduced visual performance described in myopes and identify cellular biomarkers of myopia progression that may predispose myopic eyes to myopia-associated remodeling and pathologies.

## 2. Results

### 2.1. Effect of Binocular Lens Treatment on Refraction, Eye Size and Nerve Fiber Layer Thickness

At baseline before treatment started, the two experimental groups had similar refraction (treated: 0.06 ± 0.16 D, control: 0.19 ± 0.24 D, *p* = 0.63), eye size (treated: 5.80 ± 0.03 mm, control: 5.83 ± 0.03 mm, *p* = 0.55) and ppRNFL thickness (treated: 41.83 ± 2.59 μm, control: 36.43 ± 2.32 μm, *p* = 0.17).

**Figure 1:**
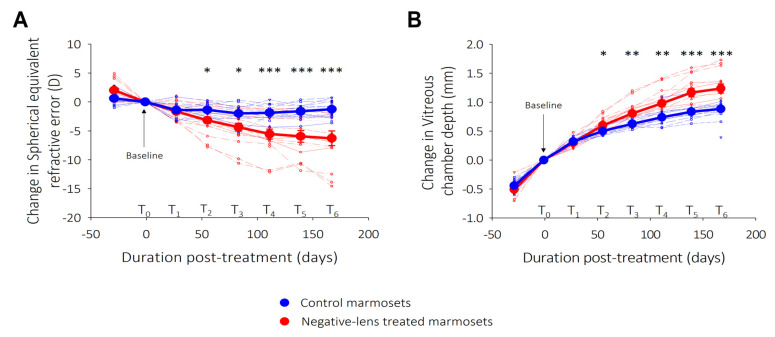
Treated marmosets developed lens-induced axial myopia. (**A**) Change in spherical equivalent refractive error and (**B**) vitreous chamber depth, normalized to baseline, as a function of treatment duration (in days). Filled circles represent the mean for each group at time points during experimental period when the refractive state and ocular biometry were assessed: control (blue) and negative-lens treated (red) marmosets. Error bars represent standard error of the mean. Statistically significant difference at each treatment time point is indicated by * *p* < 0.05, ** *p* < 0.01 and *** *p* <  0.001. Significant differences in changes in refractive error and vitreous chamber depth between treated and control marmosets are observed from T2 (8 weeks post treatment) to T6 (end of treatment).

At the end of treatment, treated and control eyes differed in refraction and vitreous chamber depth (Figure 1). The myopia that developed in negative-lens treated eyes was axial in nature and due to vitreous chamber elongation (correlation between refraction and vitreous chamber, R^2^ = 0.77, *p* < 0.001). The marmosets studied were grouped into five subcategories to perform the different analyses: ganglion cell density, astrocyte density, GFAP expression and intensity, ppRNFL thickness and inner retinal function (PhNR).

**Figure 2:**
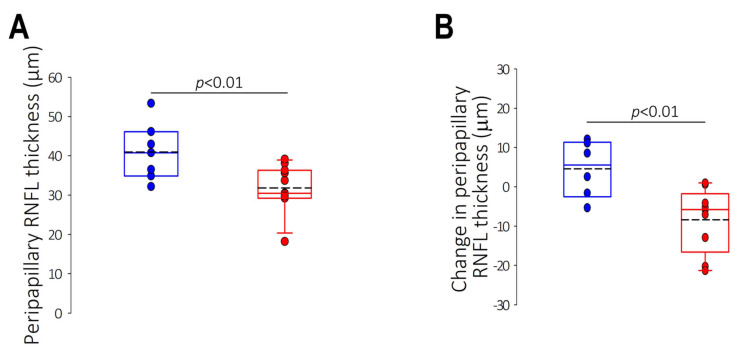

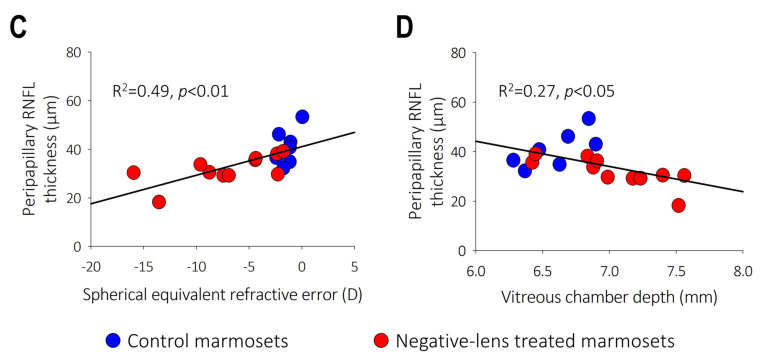
Mean peripapillary retinal nerve fiber layer (RNFL) thickness characteristics in control (blue) and negative-lens treated (red) marmosets. (**A**) Box plots illustrating treated and control marmosets’ mean peripapillary RNFL thickness at the end of treatment, and (**B**) change in peripapillary RNFL thickness (end of treatment minus baseline) sampled from a 1.73 mm radius circle from the center of the optic nerve head. (**C**) Peripapillary RNFL thickness plotted as a function of spherical equivalent refractive error and (**D**) vitreous chamber depth for treated (n = 11) and control (n = 7) marmosets. Solid black lines represent linear regression for all study marmosets.

At the end of treatment, treated marmosets had thinner ppRNFL than controls (treated: 31.82 ± 1.76 μm, control: 40.98 ± 2.76 μm, *p* < 0.01; Figure 2A), whose ppRNFL had thickened over time (change in ppRNFL thickness throughout treatment: treated: −8.34 ± 2.72 μm, control: +4.58 ± 2.93 μm, *p* < 0.01; Figure 2B); whereas the ppRNFL thinned significantly with increasing myopia (R^2^ = 0.49, *p* < 0.01; Figure 2C) and vitreous elongation (R^2^ = 0.27, *p* < 0.05, Figure 2D).

### 2.2. Characterization of Retinal Ganglion Cell (RGC) and Astrocyte Density and Distribution in the Untreated Marmoset Retina

**Figure 3:**
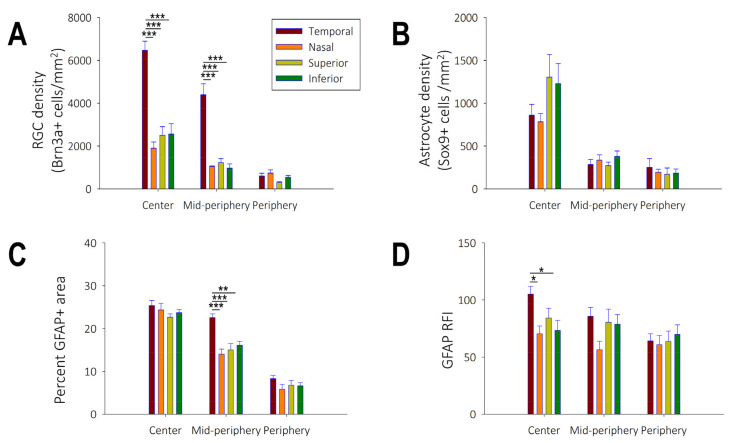
(**A**) Regional distribution of retinal ganglion density (n = 3), (**B**) astrocyte cell body density (n = 5), (**C**) glial fibrillary acidic protein (GFAP) percent coverage area (n = 10) and (**D**) relative frequency index of GFAP (n = 10) in control marmosets (N = 7) for the temporal, nasal, superior and inferior quadrants in the central, mid-peripheral and peripheral retina. Data are presented as mean  ±  standard error of the mean. N = number of marmosets; n = number of retinas. * *p* < 0.05, ** *p* < 0.01, *** *p* < 0.001.

In control marmoset eyes, ganglion cell density decreased from the center of the retina to the periphery (peripapillary: 13,417 ± 617 cells/mm^2^; mid-periphery: 7629 ± 426 cells/mm^2^; periphery: 2183 ± 374 cells/mm^2^, *p* < 0.001; Figure 3A).

**Figure 4:**
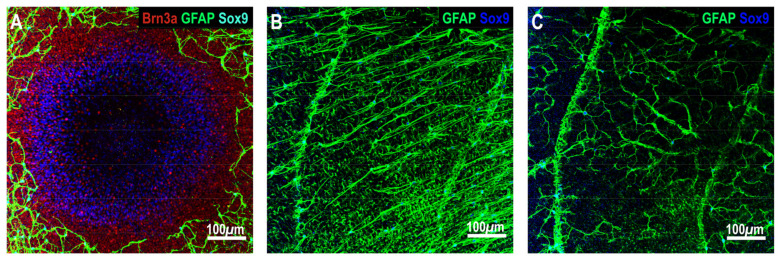
Representative confocal images of a control marmoset immunostained with Brn3a to visualize ganglion cells (red), Sox9 to visualize astrocyte cell bodies (cyan) and glial fibrillary acidic protein (GFAP) to visualize astrocyte processes (green). (**A**) Foveal and parafoveal confocal image showing the highly dense population of ganglion cells and the absence of astrocytic cell bodies and processes. Sox9+ (blue) staining in the fovea and parafoveal regions are Müller cell bodies as they are found in a deeper retinal layer (inner nuclear layer). (**B**,**C**) Astrocyte staining morphology in the central/peripapillary retina using GFAP (green; astrocytic processes) and Sox9 (blue; astrocytic cell bodies) showing (**B**) elongated and (**C**) star-shaped astrocytes in the same region at different scanning depths. All images were acquired at 20× magnification.

The greatest concentration of ganglion cells was seen in the parafoveal region (3492 ± 289 cells/mm^2^; Figure 4A). Topographically, the greatest Brn3a+ RGC density was in the temporal peripapillary region followed by the temporal mid-peripheral region, with the least density in the superior peripheral region (Figure 3A).

Similar to the distribution of Brn3a+ RGC cells, Sox9+ astrocyte cell density decreased with eccentricity (peripapillary: 4173 ± 340 cells/mm^2^; mid-periphery: 1268 ± 143 cells/mm^2^; periphery: 800 ± 226 cells/mm^2^, *p* < 0.001; Figure 3B). However, the spatial distribution of Sox9+ labelled astrocytes was different than that of ganglion cells. Astrocyte cell density was greater along the vertical meridian (superior and inferior) compared to the horizontal meridian (nasal and temporal) (vertical: 1265 ± 167 cells/mm^2^; horizontal: 821 ± 75 cells/mm^2^; *p* < 0.05). No astrocytes were identified in the marmoset retinal fovea, parafovea and ora serrata (Figure 4A). Astrocyte cell density did not differ across quadrants (Figure 3B).

GFAP expression followed a similar pattern to Sox9+ astrocyte expression, except in the mid-periphery (Figure 3C). GFAP expression decreased from the peripapillary to the mid and peripheral retina (peripapillary: 24.03 ± 0.69; mid-periphery: 16.95 ± 0.66; periphery: 6.89 ± 0.77, *p* < 0.001). In the peripapillary and peripheral retinal areas, there was no significant difference in astrocyte GFAP expression in the temporal, nasal, superior, and inferior retinal quadrants. However, in the mid-periphery, GFAP expression was greater in the temporal quadrant (*p* < 0.001, Figure 3C). We observed two distinct astrocyte morphologies in the marmoset retina: elongated astrocytes in the superficial nerve fiber layer with processes running parallel to the nerve fiber bundle (Figure 4B), and stellate astrocytes in the ganglion cell layer directly connected to blood vessels (Figure 4C). Elongated astrocytes were observed within the peripapillary to mid-periphery while stellate astrocytes were found throughout the retina.

The GFAP relative frequency index (RFI), a measure of the intensity of GFAP staining across the retina, was observed to gradually decrease from the peripapillary to the peripheral retina, although this trend was not significant (peripapillary: 83.37 ± 5.06; mid-periphery: 75.46 ± 7.26; periphery: 64.73 ± 6.01, *p* = 0.10; Figure 3D). Generally, RFI values were highest in the temporal retina, and this pattern was significant in the peripapillary retina (*p* < 0.001).

### 2.3. Effect of Myopic Eye Growth on RGC and Astrocyte Density and Distribution

**Figure 5:**
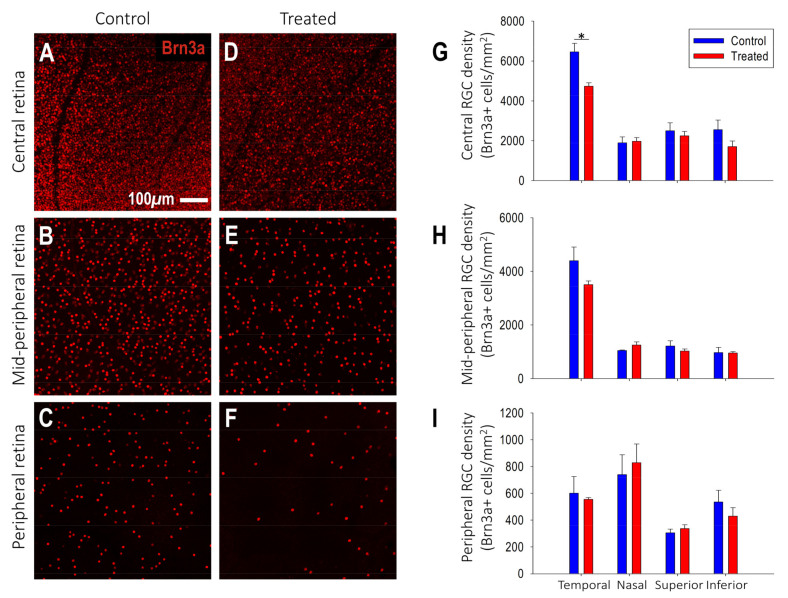
Treated myopic retinas show decreased Brn3a+ retinal ganglion cell density in the temporal central/peripapillary retinal zone compared to retinas of age-matched control marmosets. (**A**–**F**) Representative confocal images of retinal ganglion cells immunostained with Brn3a (red) acquired at 20× magnification from the (**A**,**D**) central/peripapillary, (**B**,**E**) mid-peripheral and (**C**,**F**) peripheral retinal regions of (**A**–**C**) control and (**D**–**F**) treated marmosets. (**G**–**I**) Graphs showing the differences in Brn3a+ retinal ganglion cells/mm^2^ between treated (red, n = 3) and control (blue, n = 3) marmosets for the temporal, nasal, superior, and inferior quadrants in the (**G**) central/peripapillary, (**H**) mid-peripheral and (**I**) peripheral retina. Data are presented as mean  ±  standard error of the mean. n = number of retinas. * *p* < 0.05.

Ganglion cell density: After correcting for the effect of myopic magnification, the myopic retina had a similar spatial distribution of Brn3a+ ganglion cells as controls: decreasing ganglion cell density from the center and peripapillary to the peripheral retina (Figure 5A–F). However, there was a significant difference in Brn3a+ RGC density between treated and control marmosets in the peripapillary retina (control: 13,417 ± 917 cells/mm^2^, treated: 10,668 ± 613 cells/mm^2^, *p* < 0.05; Figure 5A,D). Specifically, there was a significant reduction in Brn3a+ RGC density in the temporal peripapillary retina of myopic marmosets (≈27%) relative to age-matched controls (control: 6462 ± 124 cells/mm^2^, treated: 4739 ± 167 cells/mm^2^, *p* < 0.05; Figure 5G). Higher degrees of myopia and axial growth were associated with lower ganglion cell density in the temporal peripapillary retina (SER: R^2^ = 0.87, *p* < 0.01; VCD: R^2^ = 0.72, *p* < 0.05).

**Figure 6:**
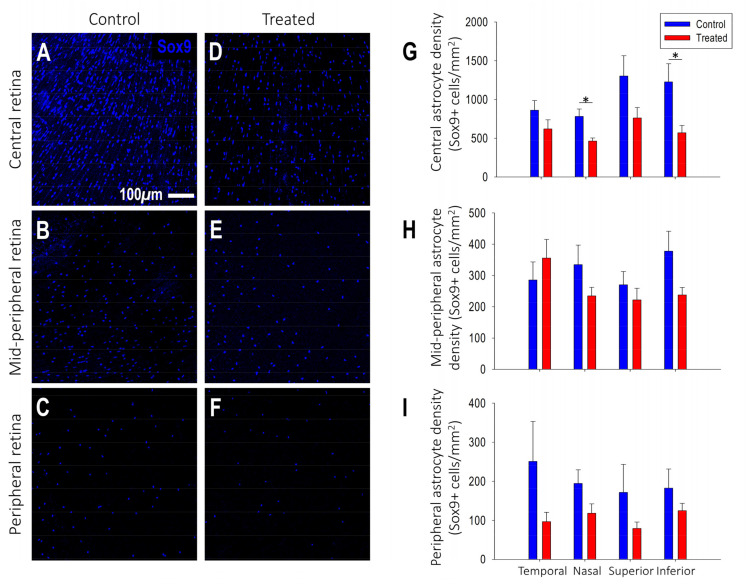
Treated myopic retinas show decreased Sox9+ astrocytic cells/mm^2^ in nasal and inferior central/peripapillary retinal zones compared to retinas of age-matched control marmosets. (**A**–**F**) Representative confocal images of astrocyte cell bodies immunostained with Sox9 (blue) acquired at 20× magnification from the (**A**,**D**) central/peripapillary, (**B**,**E**) mid-peripheral and (**C,F**) peripheral retinal regions of (**A**–**C**) control and (**D**–**F**) treated marmosets. (**G**–**I**) Graphs showing the differences in Sox9+ astrocytic cells/mm^2^ between treated (red, n = 4) and control (blue, n = 5) marmosets for the temporal, nasal, superior and inferior quadrants in the (**G**) central, (**H**) mid-peripheral and (**I**) peripheral retina. Data are presented as mean  ±  standard error of the mean. n = number of retinas. * *p* < 0.05.

Astrocyte density: The distribution pattern of decreasing astrocyte density from the peripapillary to the periphery identified in untreated controls was also present in myopic eyes (Figure 6A–F). However, treated marmosets had a lower astrocyte density compared to controls in the macula (control: 1601 ± 55 cells/mm^2^, treated: 1236 ± 152 cells/mm^2^ *p* < 0.05), nasal peripapillary (control: 782 ± 96 cells/mm^2^, treated: 463.75 ± 39 cells/mm^2^, *p* < 0.05) and inferior peripapillary retina (control: 1228 ± 234 cells/mm^2^, treated: 571 ± 95 cells/mm^2^, *p* < 0.05; Figure 6G). A significant reduction in astrocyte density was observed in the macula (R^2^ = 0.59, *p* < 0.05) and peripapillary retina (R^2^ = 0.56, *p* < 0.05) as eyes grew more myopic.

**Figure 7:**
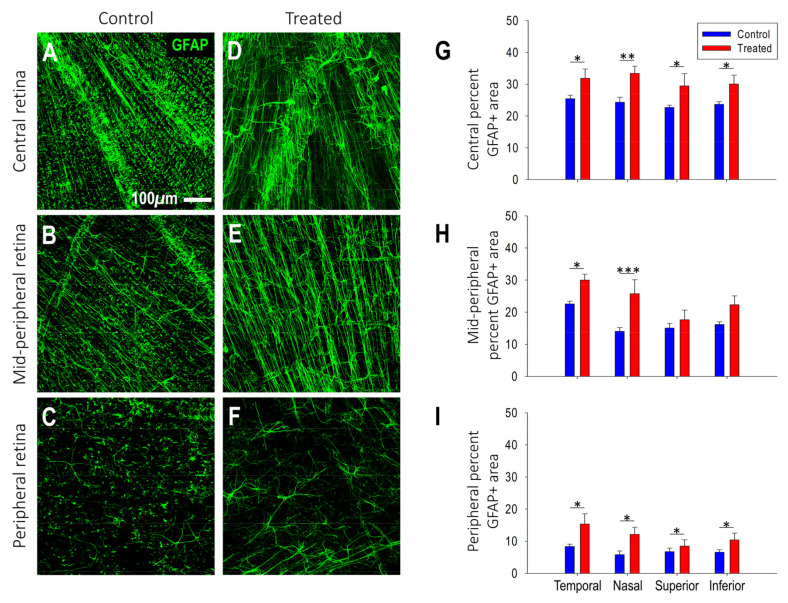
Treated myopic retinas show increased glial fibrillary acidic protein (GFAP) expression in most retinal zones and quadrants compared to retinas of age-matched control marmosets. (**A**–**F**) Representative confocal images of astrocyte cell processes immunostained with GFAP (green) acquired at 20× magnification from the (**A**,**D**) central/peripapillary, (**B**,**E**) mid-peripheral and (**C**,**F**) peripheral retinal regions of (**A**–**C**) control and (**D**–**F**) treated marmosets. (**G**–**I**) Graphs showing the differences in percentage of GFAP area coverage between treated (red, n = 6) and control (blue, n = 10) marmosets for the temporal, nasal, superior and inferior quadrants in the (**G**) central/peripapillary, (**H**) mid-peripheral and (**I**) peripheral retina. Data are presented as mean  ±  standard error of the mean. n = number of retinas. * *p* < 0.05, ** *p* < 0.01, *** *p* < 0.001.

GFAP expression: Along with a decrease in astrocyte density, treated eyes had a greater GFAP expression relative to controls in the nasal, inferior and temporal quadrants of all retina regions (peripapillary, mid-periphery and periphery). The increased GFAP expression was on average 42% greater (region specific differences are summarized in Figure 7G–I). Increased GFAP expression in areas with significant differences between the two treatment groups was associated with increased myopic growth (Supplementary Table S1). A stepwise multiple regression analysis indicated that increased GFAP expression of the nasal mid-periphery and temporal peripapillary regions was associated with at least 70% of the variations in refraction (R^2^ = 0.96; *p* < 0.001) and vitreous chamber depth (R^2^ = 0.73; *p* < 0.001) of control and myopic eyes.

The corresponding RFI for GFAP expression in the nasal mid-peripheral retina was greater in treated marmosets than controls (treated: 102.68 ± 16.42, control: 56.61 ± 7.36, *p* < 0.05) and associated with myopic growth (SER: R^2^ = 0.60, *p* < 0.01; VCD: R^2^ = 0.29, *p* < 0.05).

### 2.4. Effect of Lens Treatment on the Photopic Negative Response

No differences were observed in the ERG parameters between the two groups at the end of treatment: Vmax (treated: 121.87 ± 30.55 μV, control: 123.75 ± 30.24 μV, *p* = 0.90), K (treated: 840.42 ± 301.76 cd/m^2^, control: 838.29 ± 276.25 cd/m^2^, *p* = 0.99) or slope (treated: 0.81 ± 0.24, control: 0.76 ± 0.19, *p* = 0.66). No significant relationships were identified between PhNR Vmax and spherical equivalent refractive error (R^2^ = 0.05, *p* = 0.39) or vitreous chamber depth (R^2^ = 0.00, *p* = 0.82).

### 2.5. Relationship Between Inner Retina Structural and Functional Measures

We observed a trend towards a lower PhNR saturated amplitude as RGC density decreased, but this relationship did not reach significance (R^2^ = 0.65, *p* = 0.19). No association was observed between the PhNR and astrocyte density (R^2^ = 0.01, *p* = 0.91), GFAP expression (R^2^ = 0.01; *p* = 0.86), GFAP intensity (R^2^ = 0.05; *p* = 0.66) or ppRNFL thickness (R^2^ = 0.08, *p* = 0.24). Similarly, PhNR sensitivity and slope showed no significant relationship with any of the inner retina structural measures.

## 3. Discussion

This study identified a significant reduction in peripapillary retinal nerve fiber layer (ppRNFL) thickness, retinal ganglion cell (RGC) and astrocyte density, along with an increase in glial fibrillary acidic protein (GFAP) expression and staining intensity in the retinas of marmosets induced with myopia continuously for 6 months compared to age-matched controls. These changes remained significant after correcting for the effects of myopic magnification and occurred as myopia and eye growth increased, suggesting that the alterations in ppRNFL thickness, RGC and astrocytes distribution are likely a consequence of the growth experienced by the myopic eye as they develop increasing degrees of axial myopia. Although we observed reduced RGC and astrocyte numbers in most retinal regions, the most significant changes were localized to the peripapillary retina. Despite the significant gross-anatomical and cellular differences identified in the inner retina of myopic and control marmosets, the characteristics of the full-field photopic negative response (ffPhNR) remained unaltered and were independent of the structural differences identified.

Six months of continuous exposure to hyperopic defocus from negative lenses leads to varying degrees of compensatory changes in eye growth. Similar compensatory refractive and biometric responses to lens-imposed hyperopic defocus have been well documented in a wide range of animal species including fish [28], chicks [29], tree shrews [30], mice [31], guinea pigs [32], cats [33] and monkeys [34], and a causal relationship between axial elongation and myopia progression has been established in humans [35]. Consistent with findings from our lab, untreated control marmosets underwent emmetropization and developed low degrees of myopia and a reduction in refractive error variability [17,36]. We used an established binocular lens treatment paradigm to induce greater sustained myopic growth. This binocular lens treatment paradigm induces a greater degrees of compensation than a monocular treatment paradigm [37].

Treated marmosets had thinner peripapillary RNFL, and the thinning was associated with the degree of myopic elongation experienced. A thinner ppRNFL may be the consequence of the increased myopic eye growth experienced and due to the redistribution of RGC axons over the expanded retinal area. RGC axon stretching leading to narrower unmyelinated axons has been described in chicks with induced myopia, resulting in RNFL thinning [8,9]. However, the relationship between axial myopia and RNFL thickness in primate eyes remains equivocal. While some studies describe a significant correlation in humans between ppRNFL thickness and axial myopia [38,39], others report no relationship [40,41], or a significant correlation with only either refraction [42] or axial length [43].

While the ppRNFL did appear thinner in myopic eyes, the characteristics of the ffPhNR did not differ between treated and control animals and the ppRNFL thinning did not affect the PhNR wave. We offer two possible explanations for this observation. First, it is possible that the thinning at this stage of myopia development in marmosets does not result in detectable ffPhNR alterations. Retinal structural damage usually precedes functional loss, which suggests that corresponding functional alterations may occur later during myopia development and progression. In addition, myopic nerve fiber layer thinning tends to be asymmetric. Therefore, full-field ERGs like the ones we recorded may not have detected potential localized changes, which would be easier to identify using multifocal ERGs. Second, other cells known to contribute to the PhNR properties such as amacrine and glial cells may not be affected at this stage of myopia development. Both these hypotheses need further investigation. Lack of a relationship between retinal thickness and multifocal ERGs [16], as well as between ppRNFL thickness and full-field ERGs, has previously been reported in human myopes [25]. Conversely, retinal nerve fiber thinning from myopia development has been reported to be associated with functional retinal measures, including increased near-ultraviolet fundus reflectivity in chicks [8], as well as decreased visual field sensitivity and mean quadrant retinal response density in humans [23,44].

The pattern of Brn3a+ retinal ganglion cell distribution in the untreated control marmosets decreased in density from the peripapillary to the periphery and is consistent with the ganglion cell distribution we have previously observed in one-year-old marmosets [45]. Due to the presence of the area centralis, which has the greatest Brn3a+ ganglion cell density, marmosets exhibit a significant nasotemporal asymmetry with a steeper drop in RGC density in the temporal quadrant. Brn3a is known to be expressed by a subset of retinal ganglion cells (≈80–95%) [46], which may explain the relatively lower density we identified compared to other studies using alternative stains such as Nissl [47,48,49,50], biotin [51,52], cresyl-violet [53], and unstained retinas [54]. However, the spatial RGC distribution we report is similar to other studies on the marmoset [51,52], new world monkey (cf. *Cebus* [47], *Alouatta* [48], *Saimiri* [49], and *Aotus* [50]), old world monkey (cf. *Chlorocebus* [53], and *Macaca* [49,53]), chicks [11], bush baby [49], and human studies [49,54]. In fact, the ganglion peak density identified at the parafovea in this study is similar to that described in adult marmosets [52].

The distribution of Sox9+ astrocytes and GFAP expression and intensity across the untreated marmoset retina were fairly uniform among quadrants. Astrocyte density and GFAP expression decreased with increasing eccentricity, with density peaking in the peripapillary region. GFAP staining intensity was uniform throughout the retina. Astrocyte morphology changed from elongated (in the RNFL radial peripapillary capillary plexus) and stellate (in the ganglion cell layer superficial vascular plexus) in the peripapillary and mid-peripheral retina to only stellate in the periphery. These findings agree with earlier studies from our lab [17,45] and others on cats [55,56], macaques [55,57], and humans [58,59]. Comparatively, only star-shape astrocytes are identified in rodents [60], but their pattern of density distribution is similar to that in marmosets [61,62]. Since astrocytes guide vascular development [63], avascular retinal areas such as the fovea, parafovea and ora serrata have no astrocytes [58,64]. In humans, monkeys and cats, there is a proportional increase in astrocyte density with RNFL thickening [55,65]. This relationship between retinal astrocyte density and RNFL thickness has been described in the common marmoset previously by our lab [17,18]. Most of the GFAP immunopositive staining observed in control retinas was from astrocytes. Müller glia end feet staining assumed a distinct morphology different from that of astrocytes and did not surround Sox9+ astrocytes. We observed light GFAP+ staining of Müller cell end feet in the ganglion cell complex of control marmosets, especially in the periphery [66].

In myopic marmosets, the decrease in RGC and astrocyte density was localized to the peripapillary region. Assuming that the RGC density reduction represents cell redistribution and not cell death, a uniform density decrease across all retinal areas would have supported the existence of a uniform global expansion model of retinal stretching during myopia development. This is the case in form-deprived myopic chick eyes that exhibit a general and marked peripheral reduction in RGC density and increase in dendritic arbor [11]. However, the existence of a regional reduction in RGC density in myopic marmosets in this study may be explained by some of the different retinal expansion models that have been proposed during myopic growth. Most myopic eyes tend to grow significantly more axially than vertically or horizontally [3]. The hypotheses by Chui et al. [2] and Atchison et al. [3] proposed that either posterior or global expansion along with ganglion cell loss could explain the reduced visual performance observed in myopes. Although we did not assess visual performance and cannot determine the model of retinal expansion in the myopic marmosets studied here, non-uniform myopic growth might be the reason for the regional differences observed in cell counts [2,11]. Regional reductions in visual performance have been reported in myopes [2,3], including a decrease in acuity and resolution at 5–10° in the temporal field [2,3], which may relate to the decrease in RGC density identified in this study. The peripapillary cellular changes identified in this study may also be early markers of posterior pole changes in myopes [67]. Of particular importance among the conditions associated with myopic growth is optic neuropathy, characterized by a tilted and enlarged optic disc, and an enlargement of the temporal delta and gamma zones [68,69], which are confounding factors for the diagnosis and management of glaucoma in high myopes. Understanding these will be crucial for defining the effect and mechanism of myopia development and progression and associated myopic complications.

We postulate that the reduction in peripapillary astrocyte density, increased GFAP expression and intensity are a consequence of the effect of sustained myopic growth on retinal cell distribution. The persistent mechanical stress exerted on the retina, particularly at the posterior pole, may activate mechanosensitive ion channels, promote the release of reactive oxygen species (ROS), and induce astrocyte reactivity [70]. Astrocytes are important for the functioning of the retinal neovascular unit [71], and perturbations to their morphology and density, reflected by the upregulation of GFAP, can indicate retinal pathology [72]. The increased GFAP expression and coverage observed in treated marmosets compared to controls gave GFAP+ cells the appearance of being enlarged and the template disorganized. Due to the relationship known to exist between astrocytes and blood vessels, the reduced astrocyte density and increased GFAP expression identified in myopic marmosets may relate to vascular changes described by our lab and others including lower vessel branching, capillary regression and an increased number of string vessels, which may eventually affect ganglion cell bodies and axons [17,18]. A similar increase in GFAP expression has been recently reported in form-deprived myopic mice retinas [73]. Interestingly, the ganglion and astrocyte densities observed in myopic marmosets are similar to those observed in marmosets induced with ocular hypertension [45], which may represent early markers of ganglion cell dysfunction/loss and GFAP reactivity observed in glaucomatous marmosets [45]. A similar increase in GFAP staining intensity has been reported in glaucomatous human eyes [59].

Elongated astrocytes are densely located around the optic nerve head and are most vulnerable to increased intraocular pressure during glaucoma development [74]. Since our results describe a greater effect of myopia on the peripapillary astrocytes, this particular type of astrocyte may be preferentially affected earlier in myopia and have a detrimental effect on retinal ganglion cell axons, possibly providing a foundation for understanding myopia as a risk factor for glaucomatous optic neuropathy. Although we report of reduction in ganglion cell and astrocyte density in this study, it remains unknown whether these density changes represent cellular death. It is possible that the non-uniform retinal stretching in myopic growth increased the spacing between ganglion cells and astrocytes, subsequently displacing cells and axons to ordinal directional areas not captured in this analysis.

To understand how a greater retinal surface affects the redistribution of RGCs and astrocytes, we estimated the percentage change in RGC and astrocyte densities we would expect for the myopic growth observed and compared it to our actual results. Treated marmosets grew 3% more than controls on average (treated: 10.67 ± 0.09 mm, control: 10.35 ± 0.04 mm, *p* < 0.01), leading to an 8.19% increase in retinal surface area relative to control eyes if we model the eye as a sphere. The redistribution of RGCs and astrocytes over a retinal surface that is 8.19% greater would translate into a density decrease in myopic eyes compared to controls that would range from 3.75 to 14.16% for RGCs and 0.95 to 14.16% for astrocytes. The outcomes of our study identified an overall RGC decrease of 15.81% and 37.76% decrease in astrocyte counts per unit area in myopic eyes relative to controls. For RGCs, the actual reduction in cell density (15.81%) is close to the estimated retinal expansion decrease (3.75–14.16%), suggesting that myopic marmosets may possibly be experiencing a RGC redistribution over a larger myopic retinal area. For astrocytes, the measured global cell density reduction (37.76%) was 2.67 times the estimated retinal expansion decrease (0.95–14.16%). Due to the marked reduction in astrocyte density, the ratio of RGC/astrocyte would be increased, implying an increased load on the remaining astrocytes and decreased astrocytic support to RGC, which could lead to RGC dysfunction in long-standing myopia. This warrants further investigation.

Consistent with earlier work from our group [22], we detected no differences in the PhNR characteristics of young myopic marmosets compared to age-matched controls. We have observed attenuated inner retinal responses in marmosets within 2 weeks of treatment before marmosets developed axial myopia that disappeared over time. We hypothesized that the early functional changes identified in marmosets prior to developing myo-pia may reflect functional changes occurring as eyes adjust to the imposed defocus [54]. These functional changes in marmosets when they are exposed to a myopiagenic stimulus through the period of developing myopia highlight a complex interplay between myopia and age or its duration [17,18]. Therefore, it is likely that the adaptive mechanism in young myopes to preserve retinal function may eventually fail in long-standing myopia, leading to significant differences in retinal function measures between adult myopes and controls.

Our analysis did not identify a relationship between PhNR and ganglion cell density, astrocyte density, GFAP expression and intensity, or ppRNFL thickness. Brn3a+ ganglion cells represent a subset of the ganglion cell population in the marmoset retina [46]. Since ganglion cells are a major generator of the PhNR wave, we expected their density to be related to the intensity of the PhNR response. This lack of association may suggest that the density reduction and/or redistribution of Brn3a+ ganglion cell density at this stage of myopia development does not result in changes in PhNR. Astrocytes detect and react to mechanical stimuli in their surroundings [75], and are critical for the function and survival of retinal ganglion cells [19,76]. In ocular conditions of mechanic etiology such as the microbead occlusion model of glaucoma, there is evidence of astrocyte polarization (increased neuroprotective “A2” astrocytes and/or decreased neurotoxic “A1” astrocytes) to provide neuroprotective effects in the initial phase of the disease process [77,78]. We hypothesize that the increase in GFAP expression and intensity observed in this study may be indicative of neuroprotective astrocyte activity in the early stages of myopia development [79,80].

## 4. Materials and Methods

### 4.1. The Marmoset Myopia Model

Moderate to high myopia was experimentally induced in 14 juvenile marmosets (*Callithrix jacchus*) by treating them binocularly with negative single-vision soft contact lenses (SVSCL). Eleven age-matched marmosets were used as controls, had no lens treatment and underwent uninterrupted emmetropization. Throughout the experimental period, all marmosets were kept on a 9 h light (≈700 lux) and 15 h dark cycle as described in earlier studies by our group [22,36,81,82,83]. Food and water were available *ad libitum* with regular supplements of fruits and protein.

Treated marmosets started treatment at approx. 70 days of age and wore −5D SVSCL (diameter: 6.5 mm; base curve: 3.6–3.8 mm) in both eyes. Their degree of refractive and growth compensation to the defocus was assessed every 4 weeks. If the degree of the compensation was greater than 60% of the imposed defocus, the power of the treatment contact lenses was increased by −5D. Corneal curvature was measured with a custom-made infrared video keratometry, and lenses were fitted 0.10 mm flatter than the flattest keratometry measure. All soft contact lenses were made from methafilcon A (58% water content and 21 Dk/t, Capricornia Contact Lens, Pty Ltd., Slacks Creek, QLD, Australia). No ocular surface and anterior segment complications due to contact lens wear were detected in any marmoset enrolled in this study.

All animal procedures followed the ARVO Statement for the Use of Animals in Ophthalmic and Vision Research and were approved by the State University of New York College of Optometry Institutional Animal Care and Use Committee (IACUC).

Detailed summaries of the identification tag, assigned experiment, sex, age, refractive state, and ocular biometry raw data of control and negative-lens treated marmosets are outlined in Table 1.

### 4.2. Refractive and Ocular Biometric Measures

Refractive state and ocular biometry were assessed at baseline and every 4 weeks (4, 8, 12, 16, 20 weeks of treatment) until the end of treatment. Animals were cyclopleged using 1 drop of 1% cyclopentolate hydrochloride (Alcon Laboratories, Inc., Fort Worth, TX, USA) 20 min before measurements. On-axis refractive error was measured in awake marmosets with a Nidek ARK-900 autorefractor (Nidek Co., Ltd., Gamagori, Aichi, Japan) and converted to spherical equivalent refractive error (SER). Animals were anesthetized with alphaxalone (15 mg/kg, I.M.) prior to ocular biometry. Measurements of vitreous chamber depth (VCD) and axial length (AL) were obtained using high frequency A-scan ultrasound (25 MHz, Panametrics, NDT, Ltd., Waltham, MA, USA).

### 4.3. Peripapillary Retinal Nerve Fiber Layer Measure

Following refractive and ocular biometric measures, spectral-domain optical coherence tomography (SD-OCT) scans (Bioptigen, Inc., Durham, NC, USA) were obtained by one researcher (RKA) on anesthetized marmosets at baseline and end of treatment. The retinal scanning protocol comprises a rectangular volume retinal scan (12 × 5.40 × 12 mm^3^) composed of 700 A-scans/B-scan, 70 B-Scans (i.e., 49,000 datapoints) and 5 frames per B-scan location [36,84]. After image acquisition, the frames/B-scan were averaged to minimize speckle noise. During the OCT scans, marmosets wore rigid gas permeable contact lenses (3.75 mm base curve, 5 mm diameter, 0.00 D refraction, Conforma Laboratories, Inc., Norfolk, VA, USA), and artificial tear drops (0.5% carboxymethylcellulose sodium, Refresh Tears^®^, Allergan, Inc., Irvine, CA, USA) were also used to enhance image quality since prolonged corneal exposure and desiccation can affect image quality and the success of segmentation [85].

**Figure 8:**
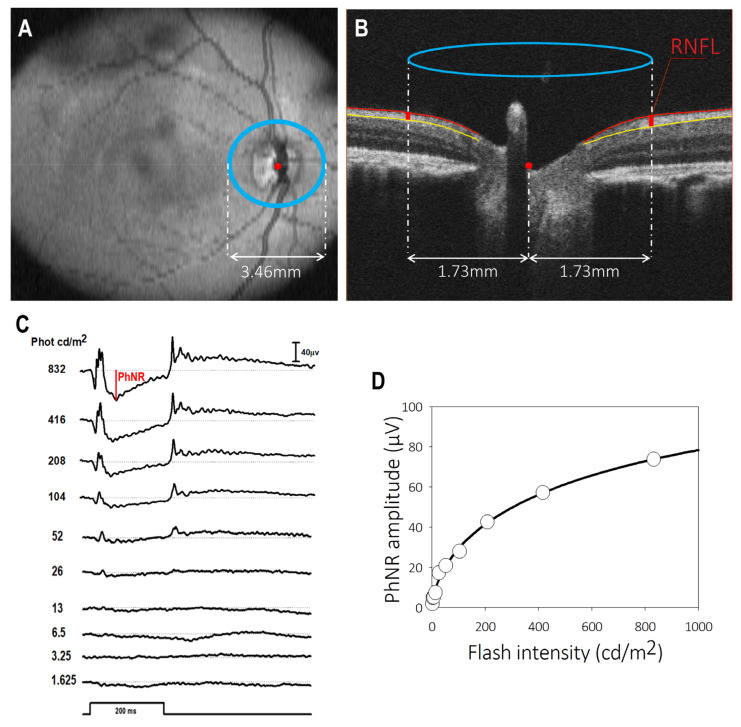
Assessment of peripapillary retinal nerve fiber layer (retinal nerve fiber layer) thickness and inner retina function (photopic negative response-PhNR) in the common marmoset. (**A**) The layout of the 1.73 mm radius (green) circle and ETDRS (white) grid for the mean peripapillary RNFL and macular thicknesses, respectively, on the OCT en face view of the marmoset eye fundus (Volume Intensity Projection image). (**B**) Automatic thickness segmentation of a cross-sectional OCT scan from a rectangular volumetric scan of a marmoset retina in XZ layout for the peripapillary retina. The center of the optic nerve head is denoted as a red dot. (**C**) Example of light-adapted ERG tracings from a common marmoset to 200 ms flashes of light of increasing intensity (1.625–832 cd/m^2^) with an illustration of the PhNR amplitude measured from baseline. (**D**) Naka Rushton equation fit for PhNR intensity–response data with the corresponding estimated saturated amplitude (Vmax), semi-saturation constant (K) and slope (*n*) parameters.

After the automatic OCT segmentation and quantification by the Iowa Reference Algorithms v3.8.0 (Retinal Image Analysis Lab, Iowa Institute for Biomedical Imaging, Iowa City, IA, USA) [86], the optic nerve head center was identified and a circle with a 1.73 mm radius was placed around it (Figure 8A). The mean peripapillary RNFL (ppRNFL) thickness was sampled along this circle (Figure 8B). We adjusted the size of the 1.73 mm radius scan circle to correct for the effect of ocular magnification [17,36].

### 4.4. Photopic Negative Response Electroretinogram Recordings

Within at most a week before the end of treatment, the light-adapted full-field photopic negative response (PhNR) in dilated anesthetized (acepromazine/ketamine, 2.5 mg/kg, 40 mg/kg, IM) marmosets was recorded using the Espion electrodiagnostic system (Diagnosys LLC, Lowell, MA, USA) as described in earlier studies [22,82]. The ERG responses were recorded to brief (200 ms) red flashes with intensities in the range of 1.56–832 cd/m^2^ on a 75 cd/m^2^ blue background. Vitals, including the body temperature, oxygen saturation, respiration and heart rate, were monitored every 5 min throughout the ERG recordings until recovery. The PhNR amplitude was measured from baseline to the PhNR wave trough (Figure 8C). Afterwards, the PhNR amplitude was plotted as a function of the flash intensity, and the data were fitted using a generalized Naka Ruston equation. The saturated amplitude (Vmax), slope, and semi-saturation constant (K) were then estimated (Figure 8D).

### 4.5. Immunohistochemistry of Whole Mounts

The immunohistochemical protocol (IHC) used in this study has been previously described [17]. Briefly, at the end of treatment, eyes were enucleated and fixed in paraformaldehyde solution (4% in phosphate-buffered saline, PBS, Santa Cruz Biotechnology, Dallas, TX, USA) for 30 min at room temperature. With special care, all extraocular muscles, periorbital fat and connective tissues were removed, and the retina was dissected. No retinal pathology was evident prior to IHC protocols. After a brief rinse in PBS (Thermo Fisher, Waltham, MA, USA), the retina was blocked in 3% normal donkey serum (NDS; Sigma-Aldrich, St. Louis, MO, USA) and 0.5% Triton X-100 (Sigma-Aldrich; for permeabilization of the cells and to reduce non-specific antibody binding) and incubated overnight at 4 °C. The retinas were then incubated with the appropriate primary antibodies for 48 h at 4 °C. The primary antibodies used for staining ganglion cells, astrocytes cell bodies and cytoskeleton were goat anti-Brn3a (1:500; Santa Cruz Biotechnology), rabbit anti-Sox9 (1:1000; Millipore Sigma, Burlington, MA, USA) and mouse anti-GFAP (1:1000; Millipore Sigma), respectively. The retinas were stained with conjugated Alexa Fluor 488-conjugated IsolectinB4 (1:100; Thermo Fisher Scientific) to assist in the identification of retinal regions and zones for analysis. The tissues were rinsed with PBS (×6; 30 min each) and incubated with fluorescent secondary antibodies for 48 h at 4 °C. The secondary antibodies used were donkey anti-goat Alexa Fluor 594, donkey anti-rabbit Alexa Fluor 488 and donkey anti-mouse Alexa Fluor 647 (at 1:500 dilution; Life Technologies, Grand Island, NY, USA). Samples were then mounted to glass slides (SuperFrost^®^ slides; Thermo Fisher) using a mounting medium with 4′, 6-diamidino-2-phenylindole (DAPI, nuclear counterstain, Vectashield; Vector Laboratories, Burlingame, CA, USA) and coverslipped. Using an Olympus FV1200 MPE confocal microscope (Tokyo, Japan) with a 20× objectives, high-resolution (1024 × 1024 pixels) retinal images were taken and analyzed quantitatively.

### 4.6. Quantification of Ganglion and Astrocytic Cell Density

**Figure 9:**
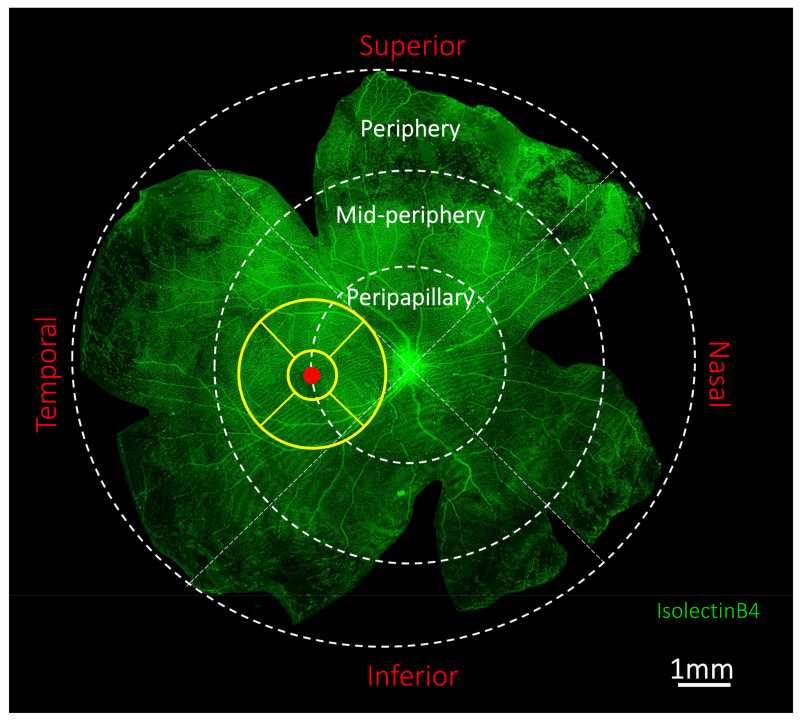
A map of the vasculature of the whole mount marmoset retina, showing the regions sampled for analysis. Retinal vasculature was visualized with conjugated IsolectinB4. Images were acquired at 4× magnification and stitched together. Yellow circles represent the innermost (foveal, 0.5 mm radius) and middle (parafoveal, 1.5 mm radius) rings of the Early Treatment Diabetic Retinopathy Study grid. Red circle indicates the fovea. White broken circles represent the central/peripapillary (<2 mm radius from optic nerve head (ONH)), mid-peripheral (2–4 mm radius from ONH), and peripheral (>4 mm radius from ONH) zones. Superior, Inferior, nasal and temporal quadrants of the retina are shown, and boundaries are indicated with white broken lines. Scale bar: 1 mm.

Retinal ganglion cell and astrocyte densities, astrocyte GFAP cytoskeletal coverage and GFAP staining intensity were quantified as previously described [17,45,80]. In summary, ganglion cells and astrocytes were identified as Brn3a labeled cells (Brn3a+) and Sox-9 stained (Sox9+) GFAP-expressing cells, respectively, at the level of the ganglion cell complex. The retina was divided into quadrants (superior, inferior, temporal and nasal) and zones (central/peripapillary: <2 mm, mid-peripheral: 2–4 mm, and peripheral: >4 mm; from the optic nerve head) using the Early Treatment Diabetic Retinopathy Study map (3 mm ETDRS: only foveal and parafoveal regions) grid (Figure 9).

The number of ganglion cells and astrocytes were manually counted and normalized to an equivalent 1 mm^2^ (corrected for each marmoset as described below) to obtain density measures for each zone, quadrant, and retina. Each region/zone was described by the average of measures from four 20 × (635.90 × 635.90 µm^2^) scans.

Myopic marmosets experience retinal stretching from axial elongation. To ensure equivalent retinal areas were analyzed in controls and treated marmosets, we corrected the retina area analyzed using the following formula:

$$A_{N}={\left(\frac{R\times AL}{9.62}\right)}^{2}$$

where *AL* is the axial length of the marmoset and *R* is length of the analysis region. The value 9.62 is the average axial length in millimeters of adult control marmosets in our lab.

Astrocytes were characterized by describing the percentage of area coverage by dividing the number of GFAP-positive pixels by the total number of pixels in the image using Fiji. We also measured the relative frequency index (RFI) by calculating the absolute difference between the maximum GFAP intensity and minimum GFAP intensity obtained from along vertical line drawn across a 3D reconstructed GFAP image.

### 4.7. Statistics

The minimum required sample sizes for each outcome variable were achieved, as determined from a power analysis (α-level = 0.05 and power = 80%) based on preliminary data from our laboratory: ganglion cell density (3 per group), astrocyte density (4 per group), GFAP expression and intensity (4 per group), ppRNFL thickness (6 per group) and inner retinal function (9 per group). Results are presented as mean ± standard error of the mean (SEM). Statistical analyses were performed using IBM SPSS Statistics for Windows (version 23.0; IBM Corp., Armonk, NY, USA). Curve fitting and graphs were made with SigmaPlot (version 10.0; Systat Software, Inc., San Jose, CA, USA). The data were assessed for normality, and parametric/non-parametric tests were used accordingly to compare measures between treated and control marmosets. Regression analyses were used to study the relationships between refractive error/vitreous chamber depth and the number of ganglion cells and astrocytes, percentage of astrocyte coverage and RFI in retinal areas where significant differences were found between the two treatment groups, as well as to explore the relationship between structural and functional measures. *p*-values less than 0.05 were considered statistically significant.

## 5. Conclusions

This study describes structural changes in NHP eyes induced with myopia, including thinning of the peripapillary RNFL, reduced ganglion cell and astrocyte density, and increased GFAP expression and intensity that remained significant after correcting for myopic magnification and were associated with increased eye growth. The PhNR did not differ between myopic and control eyes. The lack of association identified between the functional inner retinal (PhNR) and structural measures (ppRNFL thickness, the distribution and density of ganglion cells and astrocytes) in myopic marmosets suggests that the degree of non-degenerative myopia we evaluated does not elicit functional changes in ganglion cells and glia. Since we did not observe any signs of retinal degeneration in the myopic marmosets, the reduction in retinal nerve fiber layer thickness, ganglion cell and astrocyte density, increase in the expression and intensity of GFAP expression may be very early markers present prior to the development of myopia-related pathologies such as glaucoma, retinal detachment and myopic maculopathy. These findings underscore the complexity of the effect of myopic growth on inner retinal structure and function in primates, thus warranting heightened focus on deciphering the intricate mechanisms governing myopic ocular remodeling. Such insights hold profound implications for the development of targeted therapeutic interventions and clinical management strategies in combating the development and progression of this increasingly prevalent ocular disorder.

## Figures and Tables

**Table 1:** Summary of the characteristics of the study control and treated marmosets at the end of treatment.

Tag	Eye	Sex	Age (days)	SER (D)	VCD (mm)	AXL (mm)
Control marmosets (N = 11, n = 17)						
H16	LE ^d,e^	F	205	−1.12	6.61	10.31
Z18	RE ^d,e^	M	122	−1.13	6.48	10.03
Z18	LE ^e^	M	122	−1.13	6.48	10.03
H19	RE ^d,e^	M	105	−2.42	6.28	10.13
G19	RE ^d,e^	M	237	0.06	6.85	10.56
H18	RE ^c,d,e^	M	266	−2.17	6.69	10.32
H18	LE ^c^	M	266	−2.49	6.66	10.32
I18	RE ^c,d,e^	M	273	−1.14	6.63	10.29
I18	LE ^c^	M	273	−1.99	6.69	10.40
M18	RE ^c^	F	247	−1.35	6.79	10.51
M18	LE ^c,b^	F	247	−1.02	6.84	10.59
N18	RE ^c,b^	M	247	−1.26	6.59	10.28
R18	RE ^a,c,e^	F	238	−2.07	6.80	10.46
U18	RE ^a,e^	M	230	−1.16	6.83	10.24
U18	LE ^a,b,e^	M	230	−0.91	6.77	10.23
W18	RE ^c,b,d,e^	F	238	−1.08	6.90	10.61
W18	LE ^c,b^	F	238	−1.08	6.90	10.57
Mean ± SEM			222.59 ± 13.01	−1.38 ± 0.16	6.69 ± 0.04	10.35 ± 0.04
Negative-lens treated marmosets (N = 14, n = 18)						
D16	RE ^d,e^	F	336	−13.54	7.52	11.17
B17	LE ^d,e^	F	199	−7.43	7.17	10.85
E17	LE ^d,e^	F	175	−9.61	6.88	10.61
Y18	RE ^e^	F	121	−5.96	6.59	10.25
Y18	LE ^d,e^	F	121	−4.41	6.42	10.13
B19	LE ^d,e^	F	264	−2.35	6.84	10.51
C19	RE ^d,e^	F	241	−4.36	6.91	10.61
D19	RE ^e^	M	234	−2.21	6.57	10.09
D19	LE ^d,e^	M	234	−1.74	6.45	10.09
I19	LE ^d,e^	M	369	−6.91	7.23	10.89
J19	RE ^d,e^	M	369	−2.28	6.99	10.67
D18	RE ^a,c,b,d,e^	F	288	−8.76	7.40	11.02
D18	LE ^a,c,b,e^	F	288	−10.96	7.39	11.04
E19	RE ^c^	F	226	−4.18	6.52	10.31
F19	RE ^a,c,b,e^	F	233	−8.68	7.05	10.66
J18	RE ^c,d,e^	M	244	−14.89	7.49	11.11
J18	LE ^c^	M	244	−14.94	7.55	11.23
T18	LE ^b,e^	F	256	−1.89	7.09	10.74
Mean ± SEM			246.78 ± 16.34	−6.95 ± 1.05	7.00 ± 0.09	10.67 ± 0.09
Differences between groups						
	*p*-value		0.26	<0.001	<0.01	<0.01

^a^ Brn3a-stained retinas; control n = 3, treated n = 3; ^b^ Sox9-stained retinas; control n = 5, treated n = 4; ^c^ GFAP-stained retinas; control n = 10, treated n = 6; ^d^ peripapillary retinal nerve fiber layer measured retinas; control n = 7, treated n = 11; ^e^ electrical response measured retinas; control n = 11, treated n = 16; n = number of marmosets; n = number of retinas used.

## Data Availability

The datasets generated during the current study are available from the corresponding author on reasonable request.

## Supplementary Materials

The following supporting information can be downloaded at: https://www.mdpi.com/article/10.3390/ijms252413484/s1.
