# Supplementary material for: Experimental Myopia Results in Peripapillary Ganglion Cell and Astrocyte Reorganization with No Functional Implications During Early Development
Source: Int J Mol Sci. 2024 Dec 16;25(24):13484. doi: 10.3390/ijms252413484 (PMC11678399; doi:10.3390/ijms252413484)
Supplement: Supplementary file 1 [file ijms-25-13484-s001.zip › ijms-3346558-supplementary.pdf]

Supplementary Table S1. Relationship between spherical equivalent refractive error and vitreous chamber, and GFAP expression in each retinal zone (* *p*<0.05, ** *p*<0.01, *** *p*<0.001)

Retinal Zone	Refractive state (R^2, *p-value*)	Vitreous chamber depth (R^2, *p-value*)
**Nasal peripapillary**	0.42**	0.27*
**Nasal mid-periphery**	0.67***	0.53**
**Nasal periphery**	0.56***	0.37*
**Superior peripapillary**	0.45**	0.37*
**Superior mid-periphery**	0.01	0.04
**Superior periphery**	0.15	0.09
**Inferior peripapillary**	0.25*	0.22
**Inferior mid-periphery**	0.46**	0.28*
**Inferior periphery**	0.54**	0.44**
**Temporal peripapillary**	0.56***	0.49**
**Temporal mid-periphery**	0.54**	0.40**
**Temporal periphery**	0.33*	0.06
